# Does the Committee Peer Review Select the Best Applicants for Funding? An Investigation of the Selection Process for Two European Molecular Biology Organization Programmes

**DOI:** 10.1371/journal.pone.0003480

**Published:** 2008-10-22

**Authors:** Lutz Bornmann, Gerlind Wallon, Anna Ledin

**Affiliations:** 1 Professorship for Social Psychology and Research on Higher Education, ETH Zurich, Zurich, Switzerland; 2 European Molecular Biology Organization (EMBO), Heidelberg, Germany; Harvard School of Public Health, United States of America

## Abstract

Does peer review fulfill its declared objective of identifying the best science and the best scientists? In order to answer this question we analyzed the Long-Term Fellowship and the Young Investigator programmes of the European Molecular Biology Organization. Both programmes aim to identify and support the best post doctoral fellows and young group leaders in the life sciences. We checked the association between the selection decisions and the scientific performance of the applicants. Our study involved publication and citation data for 668 applicants to the Long-Term Fellowship programme from the year 1998 (130 approved, 538 rejected) and 297 applicants to the Young Investigator programme (39 approved and 258 rejected applicants) from the years 2001 and 2002. If quantity and impact of research publications are used as a criterion for scientific achievement, the results of (zero-truncated) negative binomial models show that the peer review process indeed selects scientists who perform on a higher level than the rejected ones subsequent to application. We determined the extent of errors due to over-estimation (type I errors) and under-estimation (type 2 errors) of future scientific performance. Our statistical analyses point out that between 26% and 48% of the decisions made to award or reject an application show one of both error types. Even though for a part of the applicants, the selection committee did not correctly estimate the applicant's future performance, the results show a statistically significant association between selection decisions and the applicants' scientific achievements, if quantity and impact of research publications are used as a criterion for scientific achievement.

## Introduction

Peer review is a cornerstone of science [1], [2]. It is the oldest metric used to assess scientific work by which a jury of experts is asked to evaluate the undertaking of scientific activity from an intra-scientific perspective [3], [4]. Active research scientists who are familiar with the kind of research being proposed are the best judges of the prospective impact of a research proposal on science [5]. However, critics doubt that peer review is a valid assessment instrument [6], [7]. Cole and his colleagues [8] concluded in their highly influential study on grant peer review at the National Science Foundation (NSF, Arlington, VA, USA) that “the fate of a particular application is roughly half determined by the characteristics of the proposal and the principal investigator, and about half by apparently random elements which might be characterized as ‘the luck of the reviewer draw’” (p. 885). Against this background, every scientific institution that uses peer review should ask whether the peer review system implemented fulfills its declared objective to select the best science and the best scientists. We investigated two programmes of the European Molecular Biology Organization (EMBO, Heidelberg, Germany) for the promotion and support of highly talented young scientists in the life sciences to answer this question.

Established in 1966, the Long-Term Fellowship (LTF) programme has gained an excellent reputation in the scientific community (see http://www.embo.org/fellowships/long_term.html, Access: June 12, 2008). The fellowships are awarded for a period of up to two years and are intended for advanced post doctoral research. The Young Investigator (YI) programme has been supporting outstanding young group leaders in the life sciences in Europe since 2000 (see http://www.embo.org/yip/index.html, access: June 12, 2008). The programme targets researchers who have established their first independent laboratories normally four years before the assessment in an European Molecular Biology Conference (EMBC, see http://www.embo.org/embc/, Access: September 6, 2007) member state.

The evaluation procedure for applicants to both programmes comprises of an interview with an EMBO member expert in the area of the applicant's research and an evaluation by all members of the programmes' selection committees. Each committee member individually evaluates the applicant and their research, taking into account the interviewer's report, and assigns a score between 1–10, with 10 being the best score. All applications are ranked according to their average score and decisions about approval or rejection are made after debate at a committee meeting.

To test whether indeed young scientists were selected for funding who subsequent to application developed better than the rejected ones requires a generally accepted criterion for scientific merit. The number of publications is an indicator of a scientist's research productivity. Scientific work will, if successful, result in publications [9]. An indicator for the impact of these pieces of work on the scientific community is the number of times the publications are cited in the scientific literature [10]. Both indicators provide criteria that allow us to appraise the scientific merit of the EMBO applicants [11]–[13]. We used for the evaluation the number of papers that were published by the applicants *subsequent* to application and the citations of these papers. Statistical analyses were also conducted with the citations of the papers that were published by the applicants *prior* to application. By using these standard bibliometric indicators for the analysis of the EMBO selection process, we try to answer the question, how accurately did the selection process predict the longer-term performance of a candidate [14].

Citation counts has been a controversial measure of both quality and scientific progress [15], [16]. Nevertheless, Lokker, McKibbon, McKinlay, Wilczynski, and Haynes [17] succeeded in demonstrating for clinical articles that publications regarded shortly after their appearance as important by experts in the appropriate research field were cited much more frequently in subsequent years than publications that were less highly regarded. The Chemistry Division of the NSF carried out a citation analysis with the goal “to explore the use of this relatively new tool for what it might tell about the discipline and its practitioners.” The results of the study generally support the idea that citations are meaningful [18]. Furthermore, the results of a comprehensive citation content analysis conducted by Bornmann and Daniel [19] show that “an article with high citation counts had greater relevance for the citing author than an article with low citation counts” (p. 35).

According to Evidence Ltd. – a knowledge-based company specializing in data analysis, reports and consultancy focusing on research performance – [20] “there is sufficient evidence available from experience and analysis to justify the general use of bibliometrics as an index of research performance” (p. 12).

## Methods

### Description of the dataset

**Figure 1:**
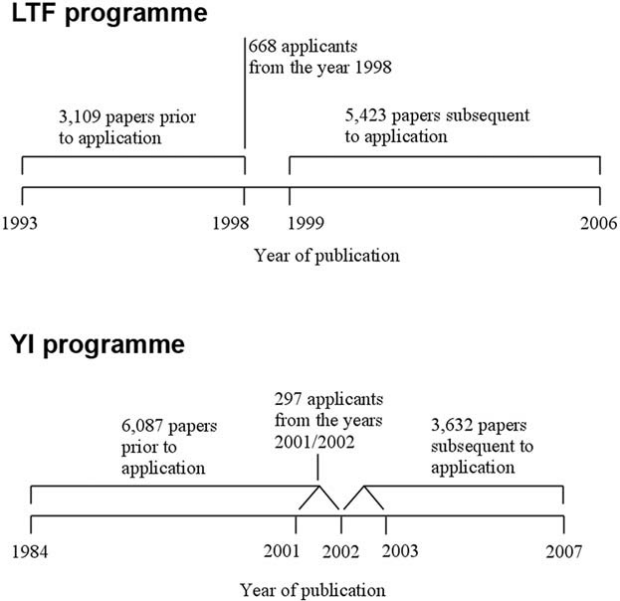
Data structure of this study.

Our study involved 668 applicants to the LTF programme from the year 1998 (130 approved, 538 rejected) (see Figure 1). Out of the total of 710 LTF applicants in the full dataset [21] we included in the present study 668 (94%); 42 withdrawn applicants were excluded. The 668 LTF applicants published a total of 3,109 papers (articles, letters, notes, and reviews) *prior* to application (publication window: from 1993 to 1998) and 5,423 papers *subsequent* to application (publication window: from 1999 to the beginning of 2006). The papers published prior to application received an average of 44.90 citations (median = 22) (according to the Science Citation Index, SCI, provided by Thomson Reuters, Philadelphia, PA, USA) and the papers published subsequent to application an average of 22.57 citations (median = 9) (citation window: from publication year until the beginning of 2006).

In addition to the applicants to the LTF programme, 297 applicants to the YI programme (39 approved and 258 rejected applicants) from the years 2001 and 2002 were included in the present study (see Figure 1). These applicants published a total of 6,087 papers (articles, letters, notes, and reviews) *prior* to application (publication window: from 1984 to the application year in 2001 or 2002) and 3,632 papers *subsequent* to application (publication window: from the application year in 2001 or 2002 to the beginning of 2007). The papers published prior to application received an average of 46.56 citations (median = 23) and the papers published subsequent to application an average of 11.15 citations (median = 4) (citation window: from publication year to the beginning of 2007).

In the citation search for the applicants' papers we included self-citations, because (1) it is not expected that the number of self-citations varies systematically for the papers published by the approved and rejected applicants, and (2) the number of self-citations of a publication can be modeled in the multiple regression analysis (the results of which are reported in the following) using the number of authors of a manuscript [22]. As Herbertz [23] shows, a greater number of authors is associated with a greater number of self-citations of a publication [24].

The bibliographic data of the applicants' papers (published prior and subsequent to application) were taken from the SCI and were double-checked in the Medline database (provided by the National Library of Medicine, NLM, Bethesda, MD, USA) and with the applicants' lists of publications. For the careful process of evaluation and cleaning, the bibliographic data were imported into a FileMaker database and matched to the information arising from the EMBO selection process (e.g., the committee's decision) [25]. To undertake the statistical analyses, two datasets (one for the LTF applicants and the other for the YIP applicants) were exported from the database to the statistical package Stata [26]. By using these datasets, the relationship between the judgments of the EMBO selection committee (approval or rejection of applications) and standard bibliometric indicators was evaluated in hindsight of the committee's decisions. In other words, we evaluated the committee's decisions with the following bibliometric indicators: (1) number of papers that were published *subsequent* to application, (2) citation counts for papers that were published *prior* and (3) *subsequent* to application.

### Statistical procedure

Bibliometric studies have demonstrated that factors other than scientific quality have a general influence on citation counts [15]: Citation counts are affected by the number of co-authors [27] and the length [28] of a paper as well as the size of the citation window [29]. That means there is a positive correlation between citation counts and the number of co-authors and the size of a paper as well as the length of the citation window. By considering these factors in the statistical analysis, it becomes possible to establish a meaningful and adjusted co-variation between decisions made by peer review and the bibliometric data gathered for the applicants.

We performed six multiple regression analyses (three for each programme), which reveal the factors that exert a primary influence on the number of papers published and citation counts. Both models predicting citation counts took the number of pages and the number of co-authors of each paper as independent variables into account besides the decision variable (dichotomous variable: 0 = rejected, 1 = approved). The publication years of the papers were included in the models predicting citation counts as exposure time [30, pp. 370–372]. We used the exposure option provided in the statistical package Stata [26] to take into account the time that a paper is available for citation. The violation of the assumption of independent observations by including citation counts of more than one paper per applicant was considered in the models by using the cluster option in Stata. This option specifies that the citation counts are independent across papers of different applicants, but are not necessarily independent within papers of the same applicant [31, section 8.3]. For each of the independent variables included in the regression models, we checked for the presence of multicollinearity by calculating variance inflation factors and tolerances [32]. The results of these analyses showed no evidences of multicollinearity.

Both outcome variables (number of papers and citations) are count variables. They indicate “how many times something has happened” [30, p. 350]. The Poisson distribution is often used to model information on counts. However, this distribution rarely fits in the statistical analysis of bibliometric data, due to overdispersion. “That is, the [Poisson] model underfits the amount of dispersion in the outcome” [30, p. 372]. Since the standard model to account for overdispersion is the negative binomial [33], we calculated in the present study negative binomial regression models (NBRMs) [34].

A second type of problem in the statistical analysis of count data occurs “when observations with outcomes equal to zero are missing from the sample because of the way the data were collected” [30, p. 381]. The statistical analysis of citation counts in the present study is based on a sample of those applicants who published at least one paper. Non-publishers were excluded, because they had not published any paper that could have been cited. Since zero-truncated count models (or zero-truncated negative binomial models, ZTNBMs) are designed for data “in which observations with an outcome of zero have been excluded from the sample” [30, p. 382], we calculated this model type if non-publishers were among the applicants in the sample (it was a necessary requirement for the model calculation to add the value 1 to each citation number to avoid zero citations).

The publication and citation data gathered for the applicants were analyzed using cycles of model specification, estimation, testing, and evaluation. We began with Poisson and then tested for negative binomial. Testing and evaluation include residual analyses and goodness-of-fit measures [35].

## Results

**Figure 2:**
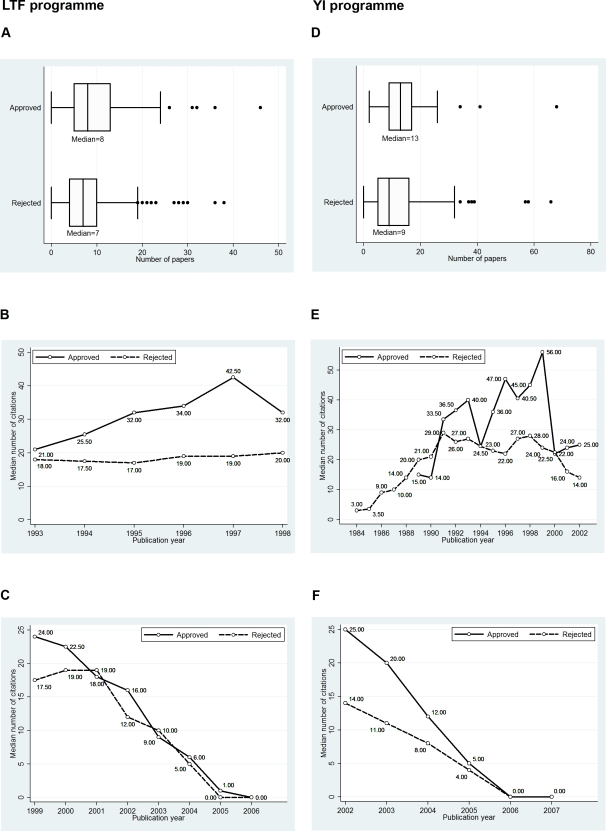
Box plots for the number of papers published subsequent to application (first row). Median numbers of citations for papers published prior to application (second row) and median numbers of citations for papers published subsequent to application (third row) (approved and rejected applicants for the LTF and YI programme). *Note*. Applications from 1998 (LTF programme) and 2001/2002 (YI programme); publication windows: from 1993 to the beginning of 2006 (LTF programme), from 1984 to the beginning of 2007 (YI programme); citation window: from year of publication to the beginning of 2006 and 2007, respectively. Since the downloading of citation counts was done in 2006 and 2007, respectively, one cannot expect high median citation counts yet for the most recent publications (see the graphs in the third row of the figure).

Did the EMBO peer review process actually achieve its goal of selecting the best young scientists? The findings in Figure 2 do not provide clear evidence that it did. The figure shows box plots for number of papers published subsequent to application (graphs A and D), univariate distributions of the median number of citations per paper per year published prior to application (graphs B and E) and univariate distributions of the median number of citations per paper per year published subsequent to application (graphs C and F). The distributions in each graph of the figure are presented separately for approved and rejected LTF and YI programmes applicants. Graph B shows, for example, that each of the papers published in 1993 by approved LTF applicants received a median of 21 citations, whereas each of the papers published in 1993 by rejected applicants received a median of 18 citations since publication until 2006. Even if in Figure 2 (1) for every publication year, the papers published by the approved LTF applicants prior to application were more often cited than papers published by the rejected applicants (graph B) and (2) approved LTF and YI applicants had published more papers subsequent to application than rejected LTF and YI applicants (graphs A and D), the median citation counts for the papers published subsequent (both programmes, graphs C and F) and prior (YI programme, graph E) to application do not demonstrate this consistent trend of an advancement for approved applicants.

### Regression analyses based on bibliometric data for the applicants to the LTF programme

Table 1 shows a description of the variables that were included in the (zero-truncated) negative binomial regression models calculated for the LTF applicants. The results of the regression analyses predicting number of papers (model 1) and citation counts (models 2 and 3) are presented in Table 2. We find that the number of pages per paper (see model 2) has a statistically significant influence on citation counts. In addition, we find that the coefficient for “Decision” is statistically significant in all three regression models. More specifically, the calculation of the percent change in expected counts [30, pp. 377–378] for a unit increase in the decision variable (from rejection to approval) following the NBRM showed that being an approved applicant increases the expected number of papers by 31%. Furthermore (see models 2 and 3), statistically significant greater numbers of citations are expected for the papers published by approved applicants prior or subsequent to applications, respectively (increased by 53% and 22%), than for the papers published by rejected applicants – holding all other variables in the models constant.

**Table 1:** Description of the factors that were potentially associated with quantity and impact of research publications (applicants for the LTF programme).

Variable	Arithmetic mean or percent	Standard deviation	Minimum	Maximum
*Model 1: Number of papers published subsequent to application (outcome variable)*				
Number of papers	8.12	6.13	0	46
Decision	20%		0 (rejected)	1 (approved)
*Model 2: Citations for papers published prior to application (outcome variable)*				
Citations (+1)	45.97	112.36	1	4,996
Decision	28%		0 (rejected)	1 (approved)
Number of pages	8.34	4.41	1	95
Number of co-authors	6.13	19.67	1	663
*Model 3: Citations for papers published subsequent to application (outcome variable)*				
Citations (+1)	23.60	43.32	1	1,123
Decision	24%		0 (rejected)	1 (approved)
Number of pages	9.21	4.53	1	78
Number of co-authors	6.88	35.78	1	2,458

**Table 2:** (Zero-truncated) negative binomial regression models predicting (1) number of papers published subsequent to application, (2) citations for papers published prior to application and (3) citations for papers published subsequent to application (applicants for the LTF programme).

	Model 1: number of papers published subsequent to application	Model 2: citations for papers published prior to application	Model 3: citations for papers published subsequent to application
Decision (1 = approved)	0.271*** (3.93)	0.422*** (4.65)	0.196* (2.09)
Number of pages		0.04*** (5.60)	0.00688 (1.14)
Number of co-authors		0.00843 (1.60)	0.0128 (1.23)
Publication year		(exposure)	(exposure)
Intercept	2.035*** (65.29)	−4.404*** (−47.17)	−4.979*** (−51.96)
*n_papers_*		3,102	5,359
*n_applicants (clusters)_*	668	652^1^	645^1^
Papers per applicant (cluster)		minimum = 1	minimum = 1
		mean = 5	mean = 8
		maximum = 28	maximum = 46
Percent change in expected counts for a unit increase in “Decision” with 95% confidence interval	31% [15%–50%]	53% [28%–82%]	22% [1%–46%]

*Note.* ML-point estimates (the results of the *z*-test in parentheses).

^*^ *p*<0.05, ^**^ *p*<0.01, ^***^ *p*<0.001.

1 truncated sample.

There is one paper in the sample for model 3 with an exorbitant number of co-authors (*n* = 2,458) (see Table 1). Omitting this paper from the regression analysis did not alter the statistically significant coefficient for the variable “Decision” that is presented in the table.

Interpretation example for the parameter estimates in the table: In model 2 the number of pages of a publication has a statistically significant effect on receiving citations with a parameter estimate of 0.04. This means that for an additional page, the odds of receiving citations increase by a factor of 1.04 ( = exp(0.04)), holding all other variables in model 2 constant.

### Regression analyses based on bibliometric data for the applicants to the YI programme

We carried out the regression analyses described above for the applicants of the YI programme. Table 3 shows a description of the variables that were included in the models. The results of the analyses are presented in Table 4. For this dataset both the page number (model 2) and the number of co-authors per paper (model 3) have statistically significant effects on citation counts. With regard to the decision of the selection committee, all three regression models yield statistically significant effects. For an approved applicant, the expected scientific mean performance is increased by 31% (number of papers), by 41% (citations for papers published *prior* to application) and by 49% (citations for papers published *subsequent* to application) against a rejected applicant, holding all other variables in the models (models 2 and 3) constant.

**Table 3:** Description of the factors that were potentially associated with quantity and impact of research publications (applicants for the YI programme).

Variable	Arithmetic mean or percent	Standard deviation	Minimum	Maximum
*Model 1: Number of papers published subsequent to application (outcome variable)*				
Number of papers	12.23	9.64	0	68
Decision	13%		0 (rejected)	1 (approved)
*Model 2: Citations for papers published prior to application (outcome variable)*				
Citations	46.57	76.70	0	1,605
Decision	14%		0 (rejected)	1 (approved)
Number of pages	8.26	4.58	1	119
Number of co-authors	5.73	11.67	1	544
*Model 3: Citations for papers published subsequent to application (outcome variable)*				
Citations (+1)	12.30	23.20	1	525
Decision	16%		0 (rejected)	1 (approved)
Number of pages	9.24	4.34	1	58
Number of co-authors	6.33	11.36	1	438

**Table 4:** (Zero-truncated) negative binomial regression models predicting (1) number of papers published subsequent to application, (2) citations for papers published prior to application and (3) citations for papers published subsequent to application (applicants for the YI programme).

	Model 1: number of papers published subsequent to application	Model 2: citations for papers published prior to application	Model 3: citations for papers published subsequent to application
Decision (1 = approved)	0.267* (2.22)	0.343*** (3.46)	0.399** (3.28)
Number of pages		0.031*** (5.32)	0.0194 (1.74)
Number of co-authors		0.0249 (1.58)	0.0416*** (3.38)
Publication year		(exposure)	(exposure)
Intercept	2.464*** (55.61)	−4.24*** (−36.73)	−6.389*** (−36.18)
*n_papers_*		6,063	3,535
*n_applicants (clusters)_*	297	297	294^1^
Papers per applicant (cluster)		minimum = 2	minimum = 1
		mean = 20	mean = 12
		maximum = 92	maximum = 65
Percent change in expected counts for a unit increase in “Decision” with 95% confidence interval	31% [3%–65%]	41% [16%–71%]	49% [17%–89%]

*Note.* ML-point estimates (the results of the *z*-test in parentheses).

^*^ *p*<0.05, ^**^ *p*<0.01, ^***^ *p*<0.001.

1 truncated sample.

Interpretation example for the parameter estimates in the table: In model 2 the number of pages of a publication has a statistically significant effect on receiving citations with a parameter estimate of 0.031. This means that for an additional page, the odds of receiving citations increase by a factor of 1.03 ( = exp(0.031)), holding all other variables in model 2 constant.

In the light of productivity and impact of research in science (paper numbers and citation counts), the EMBO selection committee is making good funding decisions for both programmes. The decisions correspond with the applicants' subsequent scientific performance. This is also true if only first and last author publications are considered as well as when we restrict our analyses to the group that we know has continued a career in academic science.

### Extent of type I and type II errors in EMBO committee peer review

Since in *every* grant or fellowship peer review process some good proposals are rejected and some bad proposals are accepted due to random error or systematic bias [36], it is instructive to calculate the extent of erroneous decisions [37]. In type I error (also called false positive error), the EMBO selection committee concluded that an applicant had the scientific potential for promotion and was approved, when he or she actually did not, as reflected in an applicant's low scientific performance subsequent to application. Type I errors lead to the *over-estimation* of the applicant's future performance, i.e. the selected applicant will perform on the same level or below the average of the rejected group. In type II error (also called false negative error), the committee concluded that an applicant did *not* have the scientific potential for promotion and was rejected, when he or she actually did as reflected in a high scientific performance subsequent to application. Type II errors lead to the *under-estimation* of the applicant's future performance, i.e. the rejected applicant will perform on the same level or above the average of the selected group [38].

In order to consider both performance measures for each applicant (paper numbers and citation counts) in the determination of the error types for the EMBO peer review process, we used the *h* index that was recently proposed by Hirsch [39]. This index is an original and simple new measure incorporating both quantity and impact of publications in *one single* number: “A scientist has index *h* if *h* of his or her *N_p_* papers have at least *h* citations each and the other (*N_p_*−*h*) papers have fewer than *h* citations each” [39, p. 16569]. A series of studies could demonstrate that a scientist's *h* index is highly correlated with his or her paper numbers and citation counts [40]. According to Hirsch [39] an *h* index of 20 after 20 years of scientific activity characterizes a successful scientist. An *h* index of 40 after 20 years of scientific activity characterizes outstanding scientists, likely to be found only at the top universities or major research laboratories and an *h* index of 60 after 20 years characterizes truly unique individuals. As the results of Bornmann and Daniel [38], [41] show, the *h* index can not only be used to measure the performance of scientists after a long career, but also that of young scientists. The authors found that the mean *h* index for successful applicants (arithmetic mean = 3.84, median = 3) for post doctoral research fellowships was statistically significantly higher than the mean *h* index for non-successful applicants (arithmetic mean = 2.72, median = 2) and that the applicants' *h* index values correlate significantly with their publication and citation numbers.

**Figure 3:**
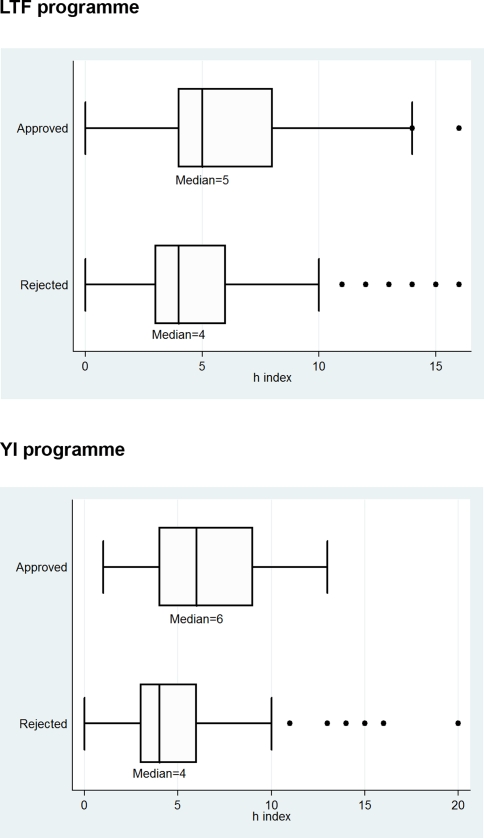
Box plots for *h* index values of approved and rejected applicants for the LTF and YI programme.

The box plots in Figure 3 show the distributions of the applicants' *h* index values. In agreement with the results reported above, the median *h* index for approved applicants is larger than that for rejected applicants, although the *h* index of both approved and rejected applicants significantly vary around the median values (see the boxes and the outliers in the figure) [42]. Among rejected applicants are scientists who have an *h* index that is higher than the median value for approved applicants, an indication of type II, i.e. false negative, errors. Among approved applicants we find scientists who have an *h* index lower than the median value for rejected applicants, an indication of type I, i.e. false positive, errors.

For the determination of the *extent* of type I and type II errors in the peer review we categorized the decision of the selection committee to approve applicants with an *h* index equal to or smaller than the median value for rejected applicants as type I error. Type II errors were defined as the rejection of applicants with an *h* index equal to or higher than the median of approved applicants (see Table 5). Based on these definitions, we calculated the extent of type I and type II errors in the peer review processes for the LTF and YI programmes. 54% (LTF programme) and 69% (YI programme) of the committee's decisions can be called correct according to our definition (see Table 6). The further percentages in the tables clearly reveal that in both programmes the selection committee made type II errors more frequently than type I errors. This means that approximately one-third of the applicants (39% and 28%) was rejected but later went on to demonstrate the same or greater scientific performance than applicants that were approved. Less than one-tenth of the applicants (7% and 3%) was approved but was subsequent not as successful as or on the same level as an “average” rejected applicant.

**Table 5:** Type I and type II errors as well as correct decisions in EMBO peer review.

Applicant's scientific output	Decision of the selection committee	
	Approval	Rejection
Applicant's scientific output is *high*	*Correct*: the *h* index is higher than the median *h* index for rejected applicants	*Type II error*: the *h* index is equal to or higher than the median *h* index for approved applicants
Applicant's scientific output is *low*	*Type I error*: the *h* index is equal to or lower than the median *h* index for rejected applicants	*Correct*: the *h* index is lower than the median *h* index for approved applicants

**Table 6:** Proportions of type I and type II errors in the decisions of the EMBO peer review for the LTF and YI programmes.

Error type	LTF programme		YI programme	
	absolute	in percent	absolute	in percent
Correct decision	362	54	204	69
Type I	48	7	10	3
Type II	258	39	83	28
Total	668	100	297	100
Errors among approvals				
Type I	48	37 (*n* = 130)	10	26 (*n* = 39)
Errors among rejections				
Type II	258	48 (*n* = 538)	83	32 (*n* = 258)

However, when interpreting the frequencies of correct and erroneous decisions, it must be taken into consideration that the extent of errors is generally dependent on the approval and rejection rates of the peer review process [38]. If the rejection rate is low, there is less risk of under-estimation, i.e. type II error. In contrast, if the approval rate is low, only few approvals are at the risk of being over-estimated, i.e. type I error. Due to scarce financial resources on one side and a large number of applicants on the other side, the present grant peer review system is especially open to type II errors [43], [44].

With approval rates of 20% (in 1998 for the LTF programme) and 13% (in 2001/2002 for the YI programme), the distributions in Table 6 are therefore hardly surprising. In order to gain an impression of the actual extent of erroneous decisions in the EMBO peer review, we included in Table 6 the proportion of type I errors within the approved group and the proportion of type II errors within the rejection group. The results show that the error rates within approved and rejected groups are between 26% and 48%, whereby again the extent of type II errors exceeds the extent of type I errors in both programme. The tables also point out that the extent of both under- and over-estimations of the applicants' scientific performance is lower for the YI programme than for the LTF programme.

## Discussion

Since “peer review can … [build,] jeopardize or destroy research efforts and careers of innovative investigators” [45, p. 34] and the advancement of scientific knowledge builds essentially on an efficient peer review system [1], the quality of each peer review process in science is of great importance. In this comprehensive study we investigated the committee peer review performed by EMBO for the selection of post doctoral fellows and young investigators. The results of the regression analyses show that the mean scientific performance of approved applicants is higher subsequent to application than the mean performance of rejected applicants. That means, there is a statistically significant association between selection decisions and the applicants' scientific achievements, if quantity and impact of research publications are used as a criterion for scientific achievement. However, as the results of the regression analyses have not been validated with independent data, there is a need for validation to generalize the findings.

In the interpretation of the results of the regression analyses it cannot be ruled out that the applicants who received funding from EMBO may have published more subsequent to application because they received funding and not necessarily because the committee made the right choice about who received funding. The higher productivity of the approved applicants against the rejected applicants may be because the committee made the right choice in deciding who should get funding but also be because they had funding allowing them (better) opportunities for research and subsequent publishing. There is circularity to this issue that should be considered in future studies investigating grant or fellowship peer review. To control in the statistical analyses for the influence of funding on subsequent publication and citation numbers, information is needed on funding of the rejected research by investigating the fate of the rejected applicants and their research projects.

Peer review processes are never faultless. With the bibliometric data of the applicants subsequent to application we were able to calculate the extent of over- and under-estimation (type 1 and type II errors) of the future success of the applicants. We find that less than one tenth of all applicants were over-estimated (approved applicants who did not perform as well as or worse than the average rejected applicant), but approximately one third were under-estimated (rejected applicants who performed equal to or above the average selected applicant). The magnitude of the under-estimation error (type II error) is a function of the success rate, i.e. scarce funding will lead to the rejection of a sizable number of worthy candidates, or reversely, an increase in success rate will reduce this error type, while increasing the risk of over-estimation (type I errors). In fact, reducing one cause for one error type (e.g., by increasing the approval rate) automatically increases the risk for the other error type. Not surprisingly both types of errors are smaller for the YI programme. 3% of the applicants have been over-estimated vs. 28% who have been under-estimated, indicating that it is easier to predict the future performance of more advanced scientists. This decrease in error rates is most likely due to the longer publication history of advanced scientists and the resulting improved view on the consistency of results produced by the scientist under evaluation.

We should also note that the applicants to the EMBO programmes are not representative of the respective post doctoral and young group leader communities at large, since they have to fulfill stringent eligibility criteria that already pre-select for high performers. Applicants to the post doctoral fellowships must have published at least one first author article in an international peer-reviewed journal, and applicants to the YI programme must have published at least one last author publication from their own independent laboratory, thereby demonstrating the ability to produce and publish independent research results. It is therefore not surprising that, given the low success rates for both programmes, the selection procedure tends to underestimate a substantial percentage of applicants.

Our review of the literature revealed that other studies on peer review also report the occurrence of errors of this kind in selection decisions. Thorngate, Faregh, and Young [44], for example, comments as follows on the grants peer review of the Canadian Institutes of Health Research (CIHR, Ottawa): “Some of the losing proposals are truly bad, but not all; many of the rejected proposals are no worse than many of the funded ones … When proposals are abundant and money is scarce, the vast majority of putative funding errors are exclusory; a large number of proposals are rejected that are statistically indistinguishable from an equal number accepted” (p. 3). According to Cole [11], the two types of errors can also take place in the journal peer review process: leaving aside speculation regarding the number of articles submitted versus available space for journal publication in the natural and social sciences, respectively, “physics journals prefer to make ‘Type I’ errors of accepting unimportant work rather than ‘Type II’ errors of rejecting potentially important work. This policy often leads to the publication of trivial articles with little or no theoretical significance, deficits which are frequently cited by referees in social science fields in rejecting articles. Other fields, such as sociology in the United States, follow a norm of rejecting an article unless it represents a significant contribution to knowledge. Sociologists prefer to make Type II errors” (p. 114).

We are aware of only four studies that investigated the quality of peer review for the selection of young scientists, only one of which included an analysis of the subsequent publication output of the applicants [46]: Melin and Danell [47] examined the peer review process for the Individual Grant for the Advancement of Research Leaders (INGVAR) of the Swedish Foundation for Strategic Research (SSF, Stockholm). Their analyses of the “publication histories” of 40 applicants show – in contrast to the results of the present study – only slight *mean* differences in scientific productivity between approved and rejected applicants. Similar results are reported by van den Besselaar and Leydesdorff [48] who evaluated the peer review process of the council for social scientific research of the Netherlands Organization for Scientific Research (Den Haag). However, the results of both studies are not directly comparable since they focused on highly selected applicants, i.e. besides the approved only the best rejected applicants. Large performance differences between accepted and rejected applicants would have been a surprise for these samples. Bornmann and Daniel [38], [41], [49] investigated committee peer review for the post doctoral fellowship programme of the Boehringer Ingelheim Fonds (B.I.F.). The authors analysed the bibliometric performance of close to 400 applicants prior to application. The results are in agreement with the findings of the present study. Hornbostel et al. [46] studied applications to the German Research Foundation's (DFG, Bonn) Emmy Noether programme. The programme funds young researchers in the late post doctoral and early group leader phase. The results show only minor differences in number of publications and citation counts between approved and rejected applicants. It can be speculated that the high success rate of applications (52%) in combination with stringent eligibility requirements have contributed to this result.

Even if the findings of this study show that the committee peer review performed by EMBO selected applicants who subsequently to selection did higher impact scientific research than rejected applicants, we still do not know whether the organisation is supporting “scientific excellence”. This question can be answered only by comparing the research performance of approved and rejected applicants with international scientific reference values [49]. Vinkler [50], [51] recommends a worldwide reference standard for the bibliometric evaluation of research groups: “*Relative Subfield Citedness* (R_w_) (where *W* refers to ‘world’) *relates the number of citations obtained by the set of papers evaluated to the number of citations received by a same number of papers … dedicated to the respective discipline, field or subfield*” (p. 164) [52]. Wuchty, Jones, and Uzzi [27] define highly cited work “as receiving more than the mean number of citations for a given field” (p. 1037), that is, with R_w_>1. Neuhaus and Daniel [53] propose for chemistry and related fields such as biology and life sciences reference values that are based on the fields/ subfields of the Chemical Abstracts database (CA, Chemical Abstracts Services, CAS, Columbus, OH, USA). In CA each paper is assigned individually to a field/ subfield. As Bornmann and Daniel [22] succeeded in applying this approach on the evaluation of the peer review process (of the journal *Angewandte Chemie-International Edition*), we will compare in a future study the publication impact of the EMBO applicants with international scientific reference values.
